# The Tim-3-Galectin-9 Pathway and Its Regulatory Mechanisms in Human Breast Cancer

**DOI:** 10.3389/fimmu.2019.01594

**Published:** 2019-07-11

**Authors:** Inna M. Yasinska, Svetlana S. Sakhnevych, Ludmila Pavlova, Anette Teo Hansen Selnø, Ana Maria Teuscher Abeleira, Ouafa Benlaouer, Isabel Gonçalves Silva, Marianne Mosimann, Luca Varani, Marco Bardelli, Rohanah Hussain, Giuliano Siligardi, Dietmar Cholewa, Steffen M. Berger, Bernhard F. Gibbs, Yuri A. Ushkaryov, Elizaveta Fasler-Kan, Elena Klenova, Vadim V. Sumbayev

**Affiliations:** ^1^Medway School of Pharmacy, Universities of Kent and Greenwich, Chatham Maritime, United Kingdom; ^2^School of Biological Sciences, University of Essex, Colchester, United Kingdom; ^3^Department of Pediatric Surgery, Department of Biomedical Research, Children's Hospital, Inselspital, University of Bern, Bern, Switzerland; ^4^Zentrum Für Medizinische Bildung, Biomedizinische Analytik HF, Bern, Switzerland; ^5^Institute for Research in Biomedicine, Universita' della Svizzera italiana, Bellinzona, Switzerland; ^6^Beamline B23, Diamond Light Source, Didcot, United Kingdom; ^7^Division of Experimental Allergology and Immunodermatology, University of Oldenburg, Oldenburg, Germany; ^8^Department of Biomedicine, University Hospital Basel and University of Basel, Basel, Switzerland

**Keywords:** galectin-9, TIM-3, breast cancer, immune evasion, immune surveillance

## Abstract

Human cancer cells operate a variety of effective molecular and signaling mechanisms which allow them to escape host immune surveillance and thus progress the disease. We have recently reported that the immune receptor Tim-3 and its natural ligand galectin-9 are involved in the immune escape of human acute myeloid leukemia (AML) cells. These cells use the neuronal receptor latrophilin 1 (LPHN1) and its ligand fibronectin leucine rich transmembrane protein 3 (FLRT3, and possibly other ligands) to trigger the pathway. We hypothesized that the Tim-3-galectin-9 pathway may be involved in the immune escape of cancer cells of different origins. We found that studied breast tumors expressed significantly higher levels of both galectin-9 and Tim-3 compared to healthy breast tissues of the same patients and that these proteins were co-localized. Increased levels of LPHN2 and expressions of LPHN3 as well as FLRT3 were also detected in breast tumor cells. Activation of this pathway facilitated the translocation of galectin-9 onto the tumor cell surface, however no secretion of galectin-9 by tumor cells was observed. Surface-based galectin-9 was able to protect breast carcinoma cells against cytotoxic T cell-induced death. Furthermore, we found that cell lines from brain, colorectal, kidney, blood/mast cell, liver, prostate, lung, and skin cancers expressed detectable amounts of both Tim-3 and galectin-9 proteins. The majority of cell lines expressed one of the LPHN isoforms and FLRT3. We conclude that the Tim-3-galectin-9 pathway is operated by a wide range of human cancer cells and is possibly involved in prevention of anti-tumor immunity.

## Introduction

Human malignant tumors have developed a variety of biochemical mechanisms which allow them to escape host immune surveillance, thus leading to disease progression (1, 2). This applies to both liquid and solid tumors (1). It has recently become evident that acute myeloid leukemia (AML) cells, originating from self-renewing myeloid haematopoietic precursors, operate an immunosuppressive pathway which includes facilitation of exocytosis of the T cell immunoglobulin and mucin domain containing protein 3 (Tim-3) and its natural ligand, galectin-9 (2–4). Galectin-9 is a tandem protein which contains two ligand-binding domains fused together by a peptide linker (several isoforms have been identified) (5). As with other galectins, galectin-9 lacks a secretory domain and thus requires trafficking in order to be translocated onto the cell surface where it could be secreted by proteolytic shedding (5). Tim-3 acts both as a receptor and a possible trafficker for galectin-9 (2, 4, 6). When associated with the plasma membrane, the Tim-3-galectin-9 complex triggers downstream signaling contributing to cell renewal, thus forming an autocrine loop (6). On the other hand, proteolytic shedding results in release of both a soluble form of Tim-3 and galectin-9 (2). Both Tim-3 and galectin-9 act to suppress anti-cancer immune surveillance (2). Secreted galectin-9 contributes to anti-cancer immune suppression by killing cytotoxic T lymphocytes and impairing the activity of natural killer (NK) cells, thus allowing for disease progression (2, 4). Soluble Tim-3 also downregulates the production of interleukin-2 (IL-2), a cytokine required for the activation of both NK cells and cytotoxic T lymphocytes (2).

AML cells express the G-protein coupled neuronal receptor latrophilin 1 (LPHN1) which is produced by haematopoietic stem cells (HSCs) but disappears upon their maturation (7, 8). In case that HSCs undergo malignant transformation, thus becoming AML cells, they preserve the ability to express LPHN1 (7, 8). Interacting with its natural ligand, fibronectin leucine rich transmembrane protein 3 (FLRT3) and possibly other ligands (2, 3), LPHN1 facilitates translocation of Tim-3 and galectin-9 onto the cell surface via GαQ [G protein transducing the signal from LPHN (7)]—phospholipase C (PLC)—protein kinase C alpha (PKCα) biochemical pathway, which is followed by proteolytic shedding of the complex or ligand-free Tim-3 (2), creating an immune suppressive “double edged sword.” Translation of Tim-3 and galectin-9 in these cells is controlled by the mammalian target of rapamycin (mTOR) pathway (2), one of the master regulators of protein biosynthesis.

Interestingly, other LPHN isoforms, in particular LPHN2, were found to be ubiquitously expressed especially in breast tumors (9). Human breast tumor cells were also found to express galectin-9, where it was shown to be involved in cell aggregation, thus preventing metastasis (10). On the other hand, the Tim-3-galectin-9 pathway plays a role in suppressing cytotoxic T cells in solid tumors, for example in colon cancer (11). However, the biochemical events underlying these processes have not been studied yet and it is therefore important to investigate whether the activity of the Tim-3-galectin-9 secretory pathway is specific solely to AML cells or whether it is also common for breast and other solid tumors. This will help us to understand the fundamental pathophysiological role of the pathway and its underlying biochemistry.

Therefore, the aim of our work was to investigate the biochemical activity of the Tim-3-galectin-9 pathway in human breast cancer and its possible role in suppressing cytotoxic T cell activity. In addition, we assessed the differential expression of the components of the Tim-3-galectin-9 pathway in human solid tumor cells.

Here we report that primary breast tumors express significantly higher levels of Tim-3, and especially galectin-9, compared to healthy tissues of the same patients. Importantly, Tim-3 and galectin-9 were co-localized. Breast tumors also expressed LPHN2 and LPHN3 as well as FLRT3. The PLC/PKC secretory biochemical pathway was significantly upregulated in breast tumors compared to healthy tissues. Breast cancer cell lines expressed all these components and biochemical studies were conducted using MCF-7 cells. Breast cancer cells were unable to secrete galectin-9, but were capable of maintaining its cell surface expression. The process of externalization was upregulated by exogenous FLRT3 most likely in a PLC/PKC-dependent manner. Surface-based galectin-9 was able to protect MCF-7 cells against T cell-induced death. Furthermore, we found that human cell lines originating from a wide range of different cancers express detectable amounts of both Tim-3 and galectin-9 proteins. The majority of these cell lines expressed at least one of the LPHN isoforms as well as FLRT3, suggesting that various types of solid and liquid tumors can in principle operate the FLRT3/LPHN/Tim-3/galecting-9 pathway.

## Materials and Methods

### Materials

RPMI-1640 medium, fetal bovine serum and supplements as well as basic laboratory chemicals were purchased from Sigma (Suffolk, UK). Maxisorp™ microtitre plates were provided either by Oxley Hughes Ltd (London, UK) or Nunc (Roskilde, Denmark). Mouse monoclonal antibodies directed against mTOR and β-actin, as well as rabbit polyclonal antibodies against phospho-S2448 mTOR, galectin-9, HRP-labeled rabbit anti-mouse secondary antibody were purchased from Abcam (Cambridge, UK). Mouse monoclonal antibody against FLRT3 was obtained from Santa Cruz Biotechnology (Heidelberg, Germany). Antibodies against phospho-S65 and total eIF4E-BP were obtained from Cell Signaling Technology (Danvers, MA USA). The polyclonal rabbit anti-peptide antibodies (PAL1, PAL2, and PAL3) against LPHN1, LPHN2, and LPHN3, respectively, were previously described (12). Rabbit polyclonal antibody against poly-(ADP-ribose)-polymerase (PAR) was purchased from Enzo Life Sciences LTD (Exeter, UK). Goat anti-mouse and goat anti-rabbit fluorescence dye-labeled antibodies were obtained from LI-COR (Lincoln, Nebraska USA). ELISA kits for the quantitation of galectin-9, Tim-3, and IL-2 were purchased from Bio-Techne (R&D Systems, Abingdon, UK). Anti-Tim-3 mouse monoclonal antibody was employed as previously described (13). Secondary antibodies for confocal laser microscopy (TRITC labeled antibody (goat anti-mouse IgG) and t FITC labeled antibody (goat anti-rabbit IgG) were from Abcam (Cambridge, UK). All other chemicals purchased were of the highest grade of purity.

### Cell Lines and Primary Human Cells

Cell lines, listed in Supplementary Table 1, were obtained from either European Collection of Cell Cultures, American Tissue Culture Collection (ATTC) or CLS Cell Lines Service GmbH. Cell lines were accompanied by identification test certificates and were grown according to corresponding tissue culture collection protocols. LAD2 mast cells were kindly provided by Prof Metcalfe and Dr. Kirshenbaum (NAID, NHI, USA) and cultured according to the protocol described before (14).

MCF-7 breast cancer cells were purchased from the European Collection of Cell Cultures (the cell lines provided were accompanied by identification test certificates). Cells were cultured in RPMI 1640 media supplemented with 10% fetal bovine serum, penicillin (50 IU/ml), and streptomycin sulfate (50 μg/ml).

TALL-104 cytotoxic ***T*** lymphocytes derived from human ***a***cute ***l***ymphoblastic ***l***eukemia (TALL) were purchased from the American Tissue Culture Collection. Cells were cultured according to the ATCC instructions. Briefly, ATCC-formulated Iscove's Modified Dulbecco's Medium was used. To make the complete growth medium we added 100 units/ml recombinant human IL-2; 2.5 μg/ml human albumin; 0.5 μg/ml D-mannitol and fetal bovine serum to a final concentration of 20%. Primary human leukocytes (PHL) were isolated from buffy coat blood (originated from healthy donors though routine blood donation). The buffy coat blood was obtained from the National Health Blood and Transfusion Service (NHSBT, UK) following ethical approval (REC reference: 16-SS-033).

### Human Tissue Samples

Primary human tumor tissue samples paired together with peripheral tissues (also called “normal” or “healthy” of the same patients) were collected surgically from breast cancer patients treated at the Colchester General Hospital, following informed, and written consent taken before surgery. Paired normal (healthy) peripheral tissues were removed during macroscopic examination of a tumor by pathologists. Blood samples were collected before breast surgery from patients with primary breast cancer (PBC) and before treatment from patients with metastatic breast cancer (MBC). Samples were also collected from healthy donors (individuals with no diagnosed pathology), which were used as control samples. Blood separation was performed using buoyancy density method employing Histopaque 1119-1 (Sigma, St. Louis, MO) according to the manufacturer's protocol. Ethical approval documentation for these studies was obtained from the NRES Essex Research Ethics Committee and the Research & Innovation Department of the Colchester Hospitals University, NHS Foundation Trust [MH 363 (AM03) and 09/H0301/37].

### Western Blot Analysis

The components of the Tim-3, galectin-9, FLRT3, LPHNs 2/3, and mTOR pathways as well as GαQ, PARP, and CD3 were detected in cell and tissue lysates by Western blot and normalized to β-actin levels in order to confirm equal protein loading as reported earlier (2). Cells were lysed in 50 mM Tris–HCl, 5 mM EDTA, 150 mM NaCl, 0.5% Nonidet-40, 1 mM PMSF, pH 8.0.

Tissue lysates for Western blot analysis were prepared as described previously (15). Briefly, 100 mg of frozen tissues were grounded into a powder in dry ice, followed by the addition of 100 μl of the tissue lysis buffer (20 mM Tris/HEPES pH 8.0, 2 mM EDTA, 0.5 M NaCl, 0.5% sodium deoxycholate, 0.5% Triton X-100, 0.25 M Sucrose, supplemented with 50 mM 2-mercaptoethanol, 50 μM PMSF, 1 μM pepstatin supplied just before use). Tissues were homogenized using a Polytron Homogenizer (Capitol Scientific, USA) and a syringe was used in order to acquire a homogenous cell suspension. These tissue suspensions were then filtered through medical gauzes and centrifuged at +4°C at 10,000 g for 15 min. Proteins present in supernatants were precipitated by incubation of the samples on ice for 30 min with equal volumes of ice-cold acetone. Protein pellets were obtained by centrifugation at +4°C, 10,000 g for 15 min followed by air drying at room temperature and then lysed using the SDS-lysis buffer described above.

Li-Cor goat secondary antibodies (dilution 1:2,000), conjugated with infrared fluorescent dyes, were used as described in the manufacturer's protocol to visualize target proteins (Li-Cor Odyssey imaging system was applied). Western blot data were quantitatively analyzed using Odyssey software and values were subsequently normalized against those of β-actin or total protein loaded.

### Enzyme-Linked Immunosorbent Assays (ELISAs)

Galectin-9, soluble Tim3 (sTim-3) and IL-2 were measured by ELISA using R&D Systems kits according to manufacturer's protocols. Phosphorylation of mTOR was analyzed by ELISA as previously described (16).

ELISA was also used to detect Tim-3-galectin-9 complex as described before (4) in the tissue homogenates. Homogenates were prepared in the ratio 1 g of tissue and 4 ml of lysis (extraction) buffer containing 50 mM Tris pH 7.5, 150 mM NaCl, 5 mM EDTA, and 0.5% NP-40. Mouse anti-Tim-3 (mAnti-Tim-3) was used as a capture antibody and biotinylated goat anti-galectin-9 (gAnti-Galectin-9, R&D Systems) for detection. The reaction was visualized using HRP-labeled streptavidin (R&D Systems; **Figure 2A**—see the scheme). In all cases plates were washed with TBST and bound secondary antibodies visualized using peroxidase reaction (ortho-phenylenediamine/H_2_O_2_).

### Quantitative Real-Time PCR (qRT-PCR)

To detect galectin-9 mRNA levels, we used qRT-PCR (4). We isolated total RNA using a GenElute™ mammalian total RNA preparation kit (Sigma-Aldrich), followed by reverse transcriptase–polymerase chain reaction (RT-PCR) of a target protein mRNA (performed according to the manufacturer's protocol). This was followed by qRT-PCR. The following primers were used: Galectin-9, 5′-CTTTCATCACCACCATTCTG-3′ and 5′-ATGTGGAACCTCTGAGCACTG-3′ actin, 5′-TGACGGGGTCACCCACACT-GTGCCCATCTA-3′, 5′-CTAGAAGCATTTGCGGTCG-ACGATGGAGGG-3′. Reactions were performed using a LightCycler^®^ 480 qRT-PCR machine and SYBR Green I Master kit (obtained from Roche, Burgess Hill, UK). The work was performed according to the manufacturer's protocol. Values representing galectin-9 mRNA levels were normalized against those of β-actin.

### On Cell Assays

On cell assays were employed to detect surface presence of galectin-9 and CD8. We used Li-Cor secondary antibody to recognize anti CD8 primary antibody and then visualized as described before (4, 17).

### Confocal Microscopy

#### Tissue Sectioning

Tissue sections were produced using a freezing microtome with the cutting thickness of 5–6 μm. Each tissue section was sliced onto a poly-D-lysine-coated microscope slide (BDH).

#### Immunofluorescence Staining for Bioimaging Analysis

Endogenous peroxidase activity was blocked by incubating slides in 3% in H_2_O_2_ for 15 min. The slides were then permeabilised using PBS containing 0.26% Triton for 20 min at room temperature and blocked with serum obtained from the same species as the secondary antibody in the following buffer: PBS, 0.05% Tween, 2% serum, 1% BSA for at least 30 min. Tim-3 and galectin-9 expressions were detected by incubating slides with antibodies described above diluted in PBS (pH 7.4 containing 0.05% Tween, 1% BSA (1:200 dilution) for 2 h at room temperature and washed three times with PBS. Slides were then incubated in the dark for 1 h with anti-IgG-FITC-labeled secondary antibody (1:400 dilution) and then washed three times with PBS followed by Fluoro-Gel mounting media containing DAPI nuclei-staining reagent. Negative controls were prepared by incubating the slides with secondary antibody alone. Images were taken using Confocal Laser Scanning Microscopy (BioRad Hercules).

#### Fluorescence Co-localization Imaging

For image acquisition, a Nikon A1si laser scanning confocal microscope was used with a Plan Fluor DIC 40x magnifying, 1.3-numerical aperture (N.A.) oil-immersion objective. NIS Elements software (version 3.21.03, Nikon, Tokyo, Japan) was employed for data analysis. Cell images were acquired in three channels for DAPI (excitation at 399 nm with laser power 10 arbitrary units [AU], emission collection at 450 nm; nuclei labeling), Alexa Fluor 488 (excitation wavelength 488 nm with laser power 10 AU and, emission wavelength at 525 nm (corresponds to a green channel, galectin-9), Alexa Fluor 555 (excitation 561 nm with laser power 10 AU, emission collection at 595 nm, red channel, Tim-3), with a photomultiplier tube gain of 100 AU. No offset was used, and pinhole size was set between 1.2 and 2 times the Airy disk size of the used objective, depending on signal strength.

### PLC and PKCα Activity Assays

The activity of PLC was measured based on the ability of this enzyme to cleave the ester bond between glycerol and phosphoric acid of the substrate phosphatidylinositol-4,5-bis-phosphate (PIP_2_). PIP_2_ (150 μM), was re-suspended in the assay buffer containing 20 mM Tris-HCl buffer (pH 7.2) containing 0.1% sodium deoxycholate, 300 μM CaCl_2_, 100 μM EDTA, and 100 mM NaCl by sonication. Reaction was started by adding the substrate followed by incubation for 60 min at 37°C. Uncleaved substrate and IP_3_ (the reaction product) were then measured using electrophoretic (33% polyacrylamide gel) separation, followed by toluidine blue staining and colorimetric assay (13, 18). The catalytic activity of PKCα was measured as described before based on its ability to phosphorylate specific substrate in a reaction buffer containing 20 mM Tris-HCl (pH7.5), 20 μM ATP, 5 mM MgCl_2_, and 200 μM CaCl_2_ (19). Phosphate groups attached to the substrate were detected spectrophotometrically (20).

### Cell Viability Assay

Cell viability was analyzed using a commercial assay kit (Promega UK Ltd., Southampton, UK). We used an MTS colorimetric assay for assessing cell metabolic activity. NAD(P)H-dependent cellular oxidoreductase enzymes playing a crucial role in human myeloid cell survival reflect the number of viable cells present. Cells were incubated with 3-(4,5-dimethylthiazol-2-yl)-5-(3-carboxymethoxyphenyl)-2-(4-sulfophenyl)-2H-tetrazolium (MTS) and then absorbance was measured at 490 nm in accordance with the manufacturer's protocol.

### Synchrotron Radiation Circular Dichroism (SRCD) Spectroscopy

Human recombinant LPHN2 (olfactomedin-like domain, MyBioSource, San Diego, CA, USA) and FLRT3, either alone or in combination (equimolar ratio), were analyzed using SRCD spectroscopy at beamline B23, Diamond Light Source (Didcot, UK) (3, 21, 22). SRCD measurements were performed using 0.7 μM of samples in a 1 cm path length cell of 3 mm aperture diameter and 60 μl capacity using a Module B instrument at 23°C. Integration time was 1 s, the increment−1 nm and bandwidth−1.2 nm. The results obtained were processed using CDApps and OriginPro^®^.

### Statistical Analysis

Each experiment was performed at least three times and statistical analysis when comparing two events at a time was conducted using a two-tailed Student's *t*-test. Multiple comparisons were performed using ANOVA. *Post-hoc* Bonferroni correction was applied. Statistical probabilities (p) were expressed as ^*^ for *p* < 0.05; ^**^ for *p* < 0.01, and ^***^ for *p* < 0.001.

## Results

### Expression and Activity of the FLRT3/LPHN/Tim3/galectin-9 Pathway in Breast Tumors

**Figure 1:**
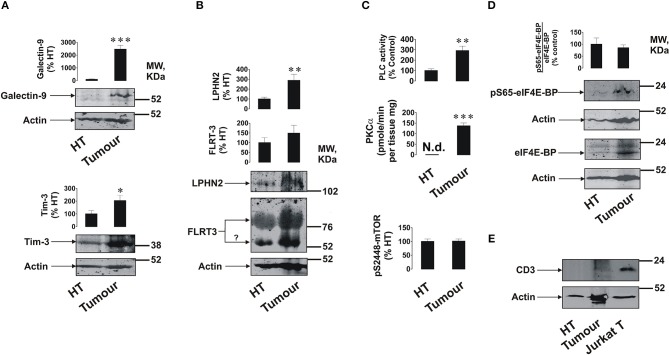
Expression of FLRT3/LPHN/Tim-3/galectin-9 pathway components and activities of PLC/PKCα and mTOR pathways in primary human breast tumors. Expression levels of Tim-3, galectin-9 **(A)**, FLRT3, and LPHN2 **(B)** were analyzed in primary breast malignant tumors and healthy breast tissues (HT) of five patients (*n* = 5) by Western blot. Activities of PLC, PKCα, and the levels of phospho-S2448 mTOR were detected as outlined in the Materials and Methods **(C)**. The amounts of phospho-S65 and total eIF4E-BP (mTOR substrate) were analyzed using Western blot **(D)**. The levels of CD3 (biomarker of T cells) were also measured using lysate of Jurkat T cells as a positive control **(E)**. Molecular weight markers (MW) are expressed in kDa. Images are from one experiment representative of five which gave similar results. Other results are shown as mean values ± SEM. ^*^p < 0.05; ^**^p < 0.01, and ^***^when *p* < 0.001 vs. control.

We found that primary breast tumors expressed galectin-9, Tim-3, LPHN2, and FLRT3 (Figures 1A,B); as well as detectable amounts of LPHN3 (Supplementary Figure 1). Interestingly, in addition to a specific FLRT3 bands, a clear band appears at around 55 kDa (highlighted by a question mark). This may represent FLRT3 which underwent proteolytic processing (3). Importantly, expression levels of Tim-3, galectin-9 and LPHN2 were significantly higher (about 15–20 fold for galectin-9, *p* < 0.001, 2 fold for Tim-3, *p* < 0.05 and 2.5–3 fold for LPHN2, *p* < 0.01) in tumors compared to healthy tissues isolated from the same patients. A band specific for galectin-9 appeared at around 55 kDa when 12% PAGE was used (this is the gel concentration normally used for galectin-9 detection) in each case and was not detectable by the anti-Tim-3 antibody. This indicates that this band is not the Tim-3-galectin-9 complex but probably a galectin-9 isoform bound to carbohydrates (as a lectin) which is unlikely to be secreted. This was confirmed when the same sample was ran using 10% PAGE and the specific band appeared above 31 kDa molecular weight marker (Supplementary Figure 2) confirming that, in a 12 % gel, protein running was “delayed” possibly due to the presence of glycosides or other post-translational modifications affecting the protein properties/shape but not the molecular weight. Activities of PLC and PKCα were significantly higher (*p* < 0.01 for PLC and *p* < 0.001 for PKCα) in tumor tissue homogenates compared to those of healthy tissues (Figure 1C). However, unlike in AML cells (2), the S2448 phosphorylation level of mTOR was similar in both healthy and tumor tissues (Figure 1C). The ratio between phospho-S65 eIF4E-BP and its total amount was also similar in both tissue types, although the amount of both phospho-S65 and total eIF4E-BP was higher in tumor tissues (Figure 1D).

Importantly, analysis of CD3 (a marker of T cells) demonstrated that this protein is undetectable in healthy and barely detectable in tumor tissue lysates suggesting that the analyzed proteins are mainly expressed by breast tumor cells and not tumor-infiltrated lymphocytes (Figure 1E).

**Figure 2:**
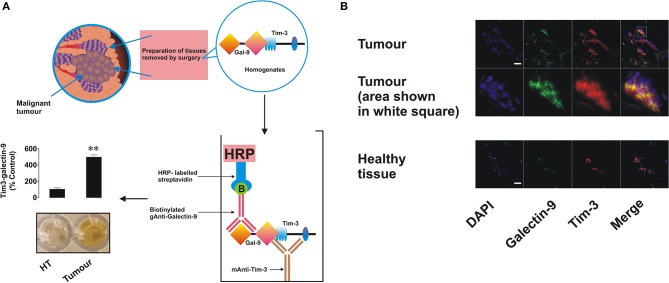
Expression, interaction, and co-localization of Tim-3 and galectin-9 in primary human breast tumors. **(A)** Presence of the Tim-3-galectin-9 complex in primary normal and tumor tissue extracts was analyzed using ELISA as outlined in the Materials and Methods. **(B)** Expression and co-localization of galectin-9 and Tim-3 were analyzed in primary human breast tumors and healthy tissues of the same patients using confocal microscopy (see Materials and Methods for further details). Images are from one experiment representative of five which gave similar results. Scale bars correspond to 20 μm.

**Figure 3:**
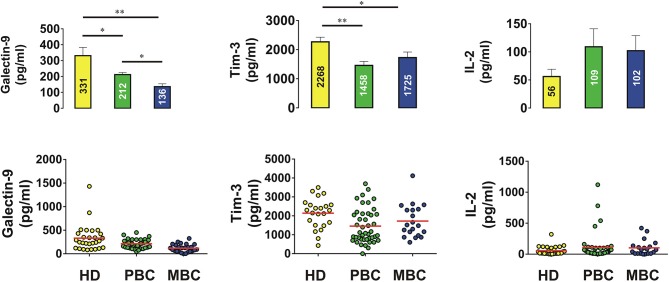
Levels of galectin-9, Tim-3, and IL-2 in blood plasma of human healthy donors and patients suffering from primary and metastatic breast tumors. Concentrations of galectin-9, soluble Tim-3, and IL-2 were analyzed in blood plasma of healthy donors and breast cancer patients by ELISA. Data are shown as mean values ± SEM of 20 for healthy donors (HD), 42 for primary breast cancer (PBC) patients, and 20 for metastatic breast cancer (MBC) patients. ^*^p < 0.05; ^**^p < 0.01 and vs. HD.

In order to assess if Tim-3 is complexed to galectin-9 as in leukemia cells, we performed detection of the Tim-3-galectin-9 complex in tissue homogenates as outlined in the Materials and Methods. We found that the complex was barely detectable in normal tissue homogenates but was clearly detectable in tumor tissue extracts (Figure 2A). Next, measurement of Tim-3 and galectin-9 in tissues was performed using confocal microscopy. In line with the data shown in Figures 1, 2A, we observed that both proteins are abundant in tumor tissue slices and are also co-localized (Figure 2B). Analysis of blood plasma samples obtained from patients with both primary and metastatic breast tumors showed that levels of galectin-9 and soluble Tim-3 were lower compared to healthy donors (Figure 3). Respectively, both patient groups demonstrated non-significantly higher levels of IL-2 (Figure 3).

**Figure 4:**
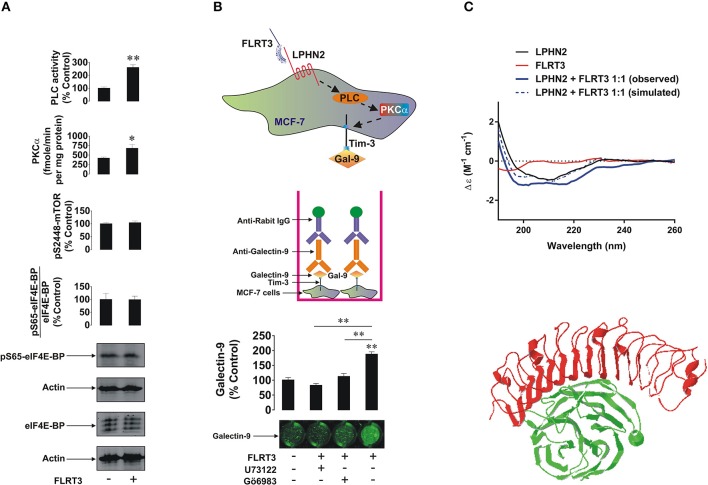
FLRT3 induces translocation of galectin-9 onto the surface of MCF-7 breast cancer cells. **(A)** MCF-7 cells were exposed for 4 h to 10 nM FLRT3 and activities of PLC, PKCα, the levels of phospho-S2448 mTOR and the amounts of phospho-S65 and total eIF4E-BP (an mTOR substrate) were analyzed as described in the Materials and Methods. **(B)** MCF-7 cells were exposed for 4 h to 10 nM FLRT3 with or without 1 h pre-treatment with 30 μM U73122 (PLC inhibitor) or 70 nM Gö6983 (PKCα inhibitor). Surface presence of galectin-9 was measured by on-cell assay. **(C)** Secondary structure and conformational changes of LPHN2 olfacomedin-like domain (FLRT3-inding region), FLRT3, and the complex of the two proteins mixed at the equimolar ratio were characterized using SRCD spectroscopy as outlined in the Materials and Methods. An interaction between olfactomedin-like domain of LPHN3 and FLRT3 generated by Swiss PDB viewer [5 cmn.pdb file downloaded through PubMed database was used (13)] is presented to illustrate the structural basis of this interaction. Images are from one experiment representative of four which gave similar results. Other results are shown as mean values ± SEM of at least three independent experiments. ^*^p < 0.05; ^**^p < 0.01 vs. control.

MCF-7, BC-8701, and MDA-MB-231 breast cancer cells (Supplementary Table 1 and Supplementary Figure 1) all expressed Tim-3, galectin-9, FLRT3, and at least one LPHN isoform. The highest level of LPHN2 was expressed by MDA-MB-231 cells, which is in line with previously reported observations (9). To further investigate the mechanism we selected MCF-7 breast cancer cells since they are the only cell line analyzed which expressed detectable amounts of both LPHNs 2 and 3 (as in primary breast tumors, Supplementary Figures 1A,C). They also expressed Tim-3 and galectin-9 (Tim-3-galectin-9 complex was also detectable, Figure 4A). Galectin-9 mRNA levels in MCF-7 cells were also significantly higher compared to normal human breast tissue (Supplementary Figure 1B). The level of galectin-9 mRNA in primary human breast tumor tissues was much higher compared to the normal tissue (Supplementary Figure 1B) and, importantly, the ratio of galectin-9 mRNA in tumor and normal tissues was similar to the respective levels of protein detected (Figure 1A). Also these results suggest that MCF-7 cells as well as primary healthy and malignant cells express identical galectin-9, thus re-confirming that the same protein was detected by Western blot (Figure 1 and Supplementary Figure 1A).

LPHN2 expression in MCF-7 cells was lower than in MDA-MB-231 but comparable with primary tumors. Furthermore, primary breast tumors and MCF-7 cells expressed comparable amounts of LPHN3 (Supplementary Figure 1C). It is important to mention that there are possible minor variations in LPHN2 gene in breast cancer cells lines (23) and possibly in primary breast tumors, however the translated protein has no variations in amino acid sequence. Both primary breast tumor and MCF-7 cells expressed GαQ (Supplementary Figure 1D), which indicates the presence of an adaptor protein required for transduction of signals *via* G-protein-coupled receptors (LPHN isoforms). Cells were also exposed for 4 h to 10 nM human recombinant FLRT3 followed by detection of phospho-S65/total eIF4E-BP, phospho-S2448 mTOR, activities of PLC and PKCα as well as cell surface presence of galectin-9. As shown in Figure 4A, exposure to FLRT3 did not affect mTOR activity but significantly upregulated the activities of PLC and PKCα. Galectin-9 surface presence was also significantly upregulated as measured by on-cell assay (Figure 4B). Importantly, pre-treatment of the cells for 1 h with 30 μM U73122 (PLC inhibitor) and 70 nM Gö6983 (PKCα inhibitor) before 4 h exposure to 10 nM FLRT3 attenuated FLRT3-induced galectin-9 translocation onto the cell surface (Figure 4B). This confirms that FLRT3-induced translocation of galectin-9 onto the surface of MCF-7 cells is controlled by the PLC/PKCα pathway.

To further verify the interaction of FLRT3 with the olfactomedin-like domain of LPHN2 [for LPHNs 1 and 3 this has already been confirmed (3, 24–26)] we performed SRCD spectroscopy analyzing spectra (thus characterizing secondary structure and conformational changes) of the olfactomedin-like domain of LPHN2 and FLRT3 alone and their equimolar combination. We found that binding of FLRT3 took place in a similar fashion as previously reported for LPHN1 (Figure 4C). This suggests that all three LPHN isoforms interact with FLRT3 in a similar way (a 3D interaction of LPHN3 oflactomedin-like domain and FLRT3 is presented in Figure 4C).

### Galectin-9 Protects Breast Cancer Cells Against Cytotoxic Immune Attack

**Figure 5:**
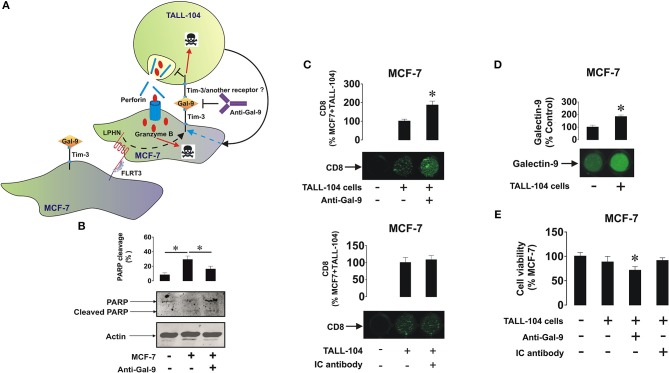
Galectin-9 protects MCF-7 cells against T cell-dependent cytotoxic immune attack. **(A)** MCF-7 cells were co-cultured with TALL-104 cytotoxic T lymphocytes at a ratio of 4 : 1 for 16 h (the ratio was determined by the aggressive behavior of TALL-104 cells) in the absence or presence of 5 μg/ml galectin-9 neutralizing antibody or 5 μg/ml isotype control antibody. **(B)** After the experiment TALL-104 cells were lysed and PARP cleavage, as an indicator of the rate of apoptotic cells, was measured using Western blot analysis. **(C)** CD8 expressions (reflecting the infiltration of TALL-104 into the MCF-7 layer) were measured by on-cell assay. **(D)** Galectin-9 surface presence was measured using on-cell assay in resting MCF-7 cells and those co-cultured with TALL-104 cells **(E)** Viability of MCF-7 cells was measured by MTS test. Images are from one experiment representative of five which gave similar results. Other results are presented as mean values ± SEM of five independent experiments. ^*^p < 0.05 vs. control.

To assess the effect of galectin-9 in protecting breast cancer cells against cytotoxic cell-dependent killing we co-cultured MCF-7 cells (adherent) with cytotoxic ALL-derived TALL-104 CD8-positive T lymphocytes (we used these rather than NK cells since mainly T cells infiltrate solid tumors; as NK cells, TALL-104 cells express Tim-3, and not galectin-9) for 16 h at a ratio of 4 MCF-7 cells: 1 TALL-104 cells (Figure 5A—this ratio was selected experimentally to achieve moderate effects in order to be able to trace biochemical mechanisms). The co-culture was performed in either the absence or presence of 5 μg/ml galectin-9 neutralizing antibody to evaluate the contribution of surface-based galectin-9. Following the treatment TALL-104 cells were collected, lysed, and subjected to Western blot analysis of full-length and cleaved PARP (marker of apoptosis). We found that in TALL-104 cells co-cultured with MCF-7 cells the level of PARP cleavage was significantly (about 3 fold, *p* < 0.05) increased and the presence of anti-galectin-9 antibody attenuated the effect (Figure 5B). Increased level of PARP cleavage indicates a higher number of apoptotic cells. An on-cell assay was used to assess the level of infiltration of TALL-104 cells into the MCF-7 cell monolayer. We observed that CD8 was absent in the MCF-7 cells when cultured on their own, but it was detected when MCF-7 cells were co-cultured with TALL-104. It was further significantly increased when the cells were co-cultured in the presence of galectin-9 neutralizing antibody, suggesting that the ability of TALL-104 cells to attack MCF-7 cells is increased when galectin-9 activity is disabled (Figure 5B). Isotype control antibody (used at the same concentration of 5 μg/ml) did not affect interactions between TALL-104 and MCF-7 cells, confirming the role of galectin-9 in this process (Figure 5C). Importantly, cell surface presence of galectin-9 was significantly upregulated in the presence of TALL-104 cells as measured by the on-cell assay (Figure 5D). Similar effect was observed before in other solid tumors (27). Viability of MCF-7 cells was also decreased in the presence of TALL-104 cells and galectin-9 neutralizing antibody (but not isotype control) as measured by an MTS test (Figure 5E). This is a strong indication that galectin-9 is capable of protecting breast tumor cells against cytotoxic cell-dependent killing.

### The Majority of Solid and Liquid Tumors Express Key Components of the FLRT3/LPHN/Tim-3/galectin-9 Pathway

**Figure 6:**
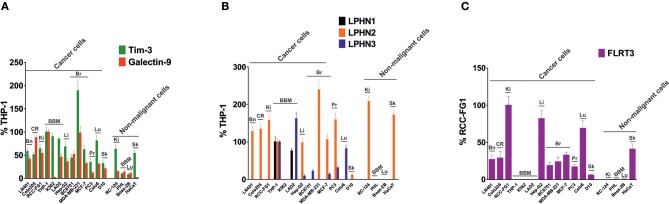
Expression of Tim-3, galectin-9, LPHNs 1, 2, and 3 as well as FLRT3 proteins in various human cancer cell lines. Lysates of indicated cells were subjected to Western blot analysis as outlined in Materials and Methods (images are presented in Supplementary Table 1). Detected infrared fluorescence of the bands divided by the total protein amounts loaded (measured using Bradford assay) was used as a measure of protein quantity. Levels of Tim-3 & total galectin-9 **(A)** and LPHNs 1, 2, & 3 **(B)** were expressed as a % of those levels present in THP-1 cells (expressed as 100%). Since THP-1 cells lack FLRT3 expression, the levels of this protein were expressed as % RCC-FG1 **(C)**, respectively considering FLRT3 level in these cells as 100%. Bn, brain; CR, colorectal; Ki, kidney; BBM, blood, bone marrow and mast cells; Li, liver; Br, breast; Pr, prostate; Lu, lung; Sk, skin. Data are presented as mean values ± SEM of three independent experiments.

To investigate the expression of FLRT3/LPHN/Tim-3/galectin-9 pathway components in cancer cells of different origins, we screened various human cancer cell lines (derived from brain, colorectal, kidney, blood/mast cell, liver, breast, prostate, lung, and skin tumors) and various non-malignant cell lines and primary cells, using Western blot analysis. Comparative analysis was performed by measuring infrared fluorescence of the bands divided by the total quantity of the loaded protein. This approach was taken because the cells analyzed originated from different tissues and thus the levels of each housekeeping protein (such as beta-actin, for example) vary depending on the origin of the cells. Results of quantitative analysis are summarized in Figure 6 and Western blot images are presented in Supplementary Table 1. Tim-3 and galectin-9 were present in all the studied cancer cells, except for the chronic myeloid leukemia (CML) cell line, K562, which expressed Tim-3 but only traces of galectin-9, in agreement with previously reported observations (2). Of note, this could be one of the reasons why CML cells entering the circulation are rapidly eliminated by cytotoxic lymphoid cells. As indicated above, we also measured the levels of secreted galectin-9 in different cells lines and observed variations dependent on their origin (Supplementary Table 1). The highest levels of galectin-9 were detected in hematological (except for K562 cells) and colorectal cancer cells. Other cell types expressed moderate levels and prostate cancer cells expressed lower but detectable levels of at least one variant of galectin-9 (Figure 6, Supplementary Table 1). Non-malignant cells, expressed lower amounts of galectin-9 and also Tim-3 compared to cancerous cells of similar origins. Furthermore, the majority of the cells expressed at least one LPHN isoform, as well as FLRT3. Some of the cells did not express FLRT3 but expressed LPHN isoforms (Figure 6, Supplementary Table 1). This most likely means that LPHN expressing cells use blood-based soluble FLRT3 to trigger the pathway since blood does not contain the other LPHN ligand teneurin-2 (7).

## Discussion

The molecular mechanisms underlying the ability of cancer cells to escape host immune surveillance remain poorly understood. Recent evidence clearly demonstrated that some tumor cells (AML in particular) operate the Tim-3-galectin-9 secretory pathway which is capable of disabling cytotoxic lymphoid cells (2, 3). However, a growing body of evidence suggests that some solid tumors [for example colorectal tumors (11)] also express Tim-3 and galectin-9 and use these proteins to escape host immune attack. We studied the activity of this pathway in breast and other solid and liquid tumors. We also investigated the pathway in both breast tumors and healthy breast tissues obtained from the same patients as well as in breast tumor cell lines.

Using Western blot analysis and confocal microscopy we found very low levels of galectin-9 in human breast tissue cells peripheral to tumor (Figures 1, 2), which were significantly increased in tumor cells. Importantly, as in AML cells, Tim-3, and galectin-9 are co-localized in breast tumor cells and are capable to form complex (Figure 2A and Supplementary Figure 1A). However, this tumor-associated galectin-9 is unlikely to be secreted since blood plasma levels of galectin-9 are lower than in healthy donors. Interestingly, high levels of Tim-3 are known to be expressed by solid tumor-infiltrating lymphocytes (27, 28), which could be used by tumor-derived galectin-9 to kill them. Our results have confirmed that Tim-3 and galectin-9 are expressed mainly by tumor cells, since CD3 (a T cell biomarker) was barely detectable in tumor and undetectable in healthy tissue lysates (Figure 1E). Furthermore, both proteins are co-localized suggesting that they are expressed by the same cells (Figure 2B). Levels of soluble Tim-3 were also downregulated in blood plasma of breast cancer patients which is in line with galectin-9 values. Importantly, as indicated above, our results indicate that galectin-9 is unlikely to be secreted by breast tumors, since its levels do not increase in patient blood plasma and the studied human breast cancer cell lines were incapable of secreting galectin-9. Furthermore, the molecular weight of tissue-based galectin-9 suggests that it is most likely associated with plasma membrane-based glycosides, as previously described in somatic cells (29–31). This binding would of course keep it attached to the cell surface and prevent its release.

Taken together, these results suggest that the FLRT3/LPHN/Tim-3/galectin-9 pathway possibly functions mainly to transfer galectin-9 onto the cell surface rather than to secrete it as in the case with AML cells. This would explain the lower level of mTOR pathway activation in breast tumor cells compared to AML cells (2), where secretion takes place and subsequently requires replacement of the proteins through biosynthesis. Interestingly, the findings reported here on the protein level are in line with gene expression data for human breast cancer presented in The Cancer Genome Atlas.

Remarkably, the level of IL-2 was non-significantly increased in blood plasma of breast cancer patients, but not in AML patients, including those with primary and metastatic breast tumors. This suggests that induction of the cytotoxic activity of NK cells and cytotoxic T cells can still take place. This observation is supported by the fact that plasma levels of soluble Tim-3 are also lower, since Tim-3 was shown to downregulate IL-2 secretion by specialized T cells (2).

Tumor tissue cells expressed LPHN2 (significantly higher levels compared to healthy tissues), LPHN3, and FLRT3 (LPHN ligand). In line with this, the activities of PLC and PKCα were significantly higher in tumors compared to healthy tissues. The activity of mTOR was not upregulated, although basal levels of the mTOR substrate (eIF4E-BP) were significantly higher in tumor cells.

**Figure 7:**
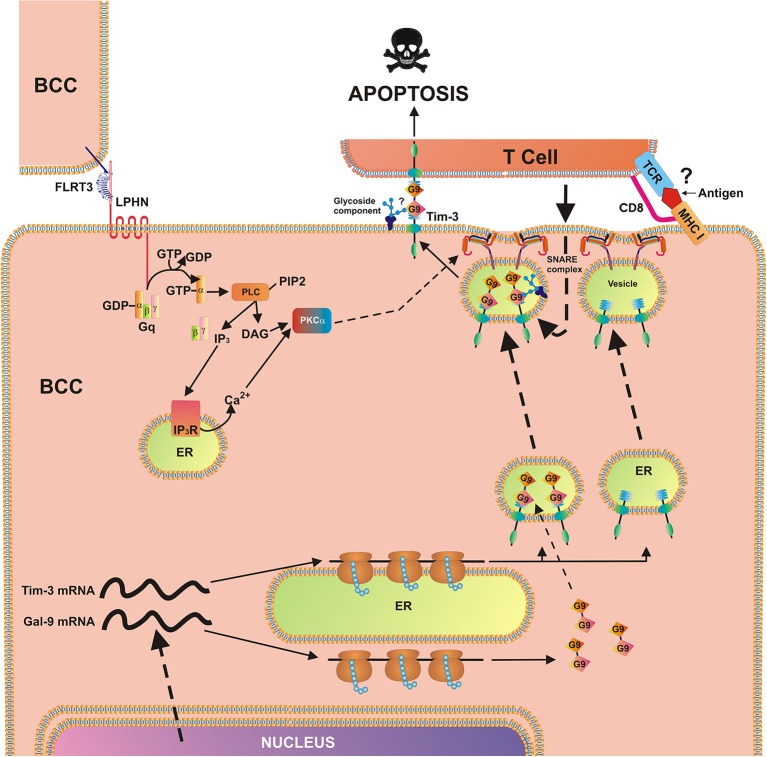
Breast cancer cell-based pathobiochemical pathways showing LPHN-induced activation of PKCα, which triggers the translocation of Tim-3 and galectin-9 onto the cell surface which is required for immune escape. The interaction of FLRT3 with LPHN isoform leads to the activation of PKCα, most likely through the classic Gq/PLC/Ca^2+^pathway. Ligand-bound LPHN activates Gq, which in turn stimulates PLC. This leads to phosphatidyl-inositol-bisphosphate (PIP_2_) degradation and production of inositol-trisphospate (IP_3_) and diacylglycerol (DAG). PKCα is then activated by DAG and cytosolic Ca^2+^. PKCα provokes the formation of SNARE complexes that tether vesicles to the plasma membrane. Galectin-9 impairs the cancer cell killing activity of cytotoxic T cells (and other cytotoxic lymphocytes). Possible (not directly confirmed) interactions of galectin-9 with glycoside component and T cell receptor (TCR)/CD8, with MHC I, and antigen are highlighted with question mark “?” to indicate the fact that it is a hypothetic interaction, since TALL-104 cells used in the study kill tumor cells in MHC-independent manner (32).

We found that exposure of MCF-7 breast cancer cells to 10 nM FLRT3 induces activation of PLC and PKCα. Recently it has been found that FLRT3 activates the PKCα pathway *via* LPHN isoforms (2). The activity of mTOR was not increased after 4 h of exposure to FLRT3. FLRT3-induced externalization of galectin-9 onto the cell surface was observed. This is in line with previous observations made in AML cells where galectin-9 secretion was shown to be triggered by LPHN in a PKCα-dependent manner (2). Importantly, in both leukemia (2) and MCF-7 cells FLRT-3-induced effects are moderate. The effects observed with latrotoxin (a LPHN1 ligand) in AML cells are much stronger compared to those of FLRT3 (2). These moderate effects indicate that they are most likely continuous and thus keep the pathway operating in order to protect malignant cells against cytotoxic lymphoid cells on an ongoing basis but in a manner that does not allow exhaustion of the cell. On the other hand, there are clearly other pathways operated by breast cancer cells to suppress the activity of cytotoxic immune cells. For example presence of TALL-104 cells significantly upregulates galectin-9 surface presence in MCF-7 breast cancer cells. Biochemical pathways underlying this phenomenon remain to be identified. Based on these observations, it can be concluded that the FLRT3-LPHN-Tim-3-galectin-9 pathway is functional in other cancer cells and in particular in breast cancer cell lines and primary tumors. In this case, however, we do not observe a FLRT3-LPHN-dependent activation of mTOR but rather a moderate, yet significant, activation of PLC/PKCα. In contrast, FLRT3-LPHN1 interactions in AML cells are capable of activating the mTOR pathway. The biological effect of LPHN2-FLRT3 results in the translocation of the Tim-3-galectin-9 complex onto the cell surface but since there is no proteolytic shedding/secretion, constant protein renewal is not required as it is in AML cells. A scheme representing the involvement of FLRT3-LPHN and other possible interactions in the activation of Tim-3-galectin-9 immunosuppressive pathway is shown in Figure 7. Intriguingly, in line with our observations that galectin-9 levels in PC3 prostate cancer cells are low compared to other cancer cells, these cells were recently reported to be rapidly killed by TALL-104 cells used in our work (32).

Interestingly, it has been previously reported that galectin-9 demonstrates anti-metastatic potential in breast cancer (10, 33). Possible reasons underlying such an activity were suggested to be galectin-9-induced cell aggregation and reduced adhesion of breast cancer cells to the extracellular matrix (10). These studies support our hypothesis regarding the possible interaction of galectin-9 with membrane-associated glycosides, since this process is known to participate in determining the membrane potential (30, 31, 34), which is a crucial factor affecting cell aggregation. Importantly, as a result of alternative splicing, galectin-9 may be present in three main isoforms characterized by the length of the linker peptide: long (49 amino acids), medium (27 amino acids) and short (15 amino acids) (5, 29–31, 33, 34). As one can see from the data reported in Supplementary Table 1, some of the cell types can express only one isoform, others two, or all three but the biochemical reasons underlying this phenomenon remain to be understood.

Additionally, NK cells interacting with galectin-9 on the breast cancer cell surface may release interferon gamma (IFN-γ) in response (2, 35) which could activate cytotoxic lymphoid cells located in the area of the tumor microenvironment. These cells can attack and kill malignant cells breaking off from the tumor thus preventing their circulation and metastasis. Overall, it appears that galectin-9 could protect the tumor which produces it in order to evade host immune attack, but this may not promote metastasis.

## Conclusion

Our findings demonstrate the activity of the Tim-3-galectin-9 biochemical pathway in breast and various other types of human cancer cells and its possible implication in suppression of host anti-cancer immune surveillance. This pathway can be recommended for targeting in order to design novel anti-cancer immunotherapeutic approaches based on inhibiting the Tim3-galectin-9 pathobiochemical pathway thus enabling the immune system to attack and eradicate malignant tumors.

## Data Availability

The datasets used and/or analyzed during the current study are available from the corresponding author on reasonable request.

## Ethics Statement

Ethical approval documentation for these studies was obtained from the NRES Essex Research Ethics Committee and the Research & Innovation Department of the Colchester Hospitals University, NHS Foundation Trust [MH 363 (AM03) and 09/H0301/37]. Buffy coat blood was purchased from the National Health Blood and Transfusion Service (NHSBT, UK) following ethical approval (REC reference: 16-SS-033).

## Author Contributions

IY, SS, and LP generated most of the data presented in the Figures 1–6. AT, MM, AMT, IG, SS, and IY generated the data presented in the Figure 6 and Supplementary Table 1. OB generated some of the data reported in the Figure 4. LV and MB generated the anti-Tim-3 antibody. DC and SB provided critical advise and contributed to manuscript writing and data interpretation. BG helped with experiments using primary human samples and contributed to manuscript writing. RH and GS together with SS, OB, IY, and VS performed SRCD spectroscopy and performed data analysis. YU developed anti-LPHN antibodies. EF-K designed the experiments using cell lines, wrote the manuscript. EK designed experiments using primary human samples, obtained primary human samples, and wrote the manuscript. VS designed the study, supervised the process of data analysis and interpretation, organizations of figures and tables, experimental design, and wrote the manuscript.

### Conflict of Interest Statement

The authors declare that the research was conducted in the absence of any commercial or financial relationships that could be construed as a potential conflict of interest.
