# Supplementary material for: The Tim-3-Galectin-9 Pathway and Its Regulatory Mechanisms in Human Breast Cancer
Source: Front Immunol. 2019 Jul 11;10:1594. doi: 10.3389/fimmu.2019.01594 (PMC6637653; doi:10.3389/fimmu.2019.01594)
Supplement: Supplementary file 1 [file Data_Sheet_1.docx]

**SUPPLEMENTARY TABLES AND FIGURES**

**The Tim-3-galectin-9 pathway and its regulatory mechanisms in human breast cancer**

Inna M. Yasinska, Svetlana S. Sakhnevych, Ludmila Pavlova, Anette Teo Hansen Selnø, Ana Maria Teuscher Abeleira, Ouafa Benlaouer, Isabel Gonçalves Silva, Marianne Mosimann, Luca Varani, Marco Bardelli, Rohanah Hussain, Giuliano Siligardi, Dietmar Cholewa, Steffen Berger, Bernhard F. Gibbs, Yuri A. Ushkaryov, Elizaveta Fasler-Kan, Elena Klenova, Vadim V. Sumbayev

**
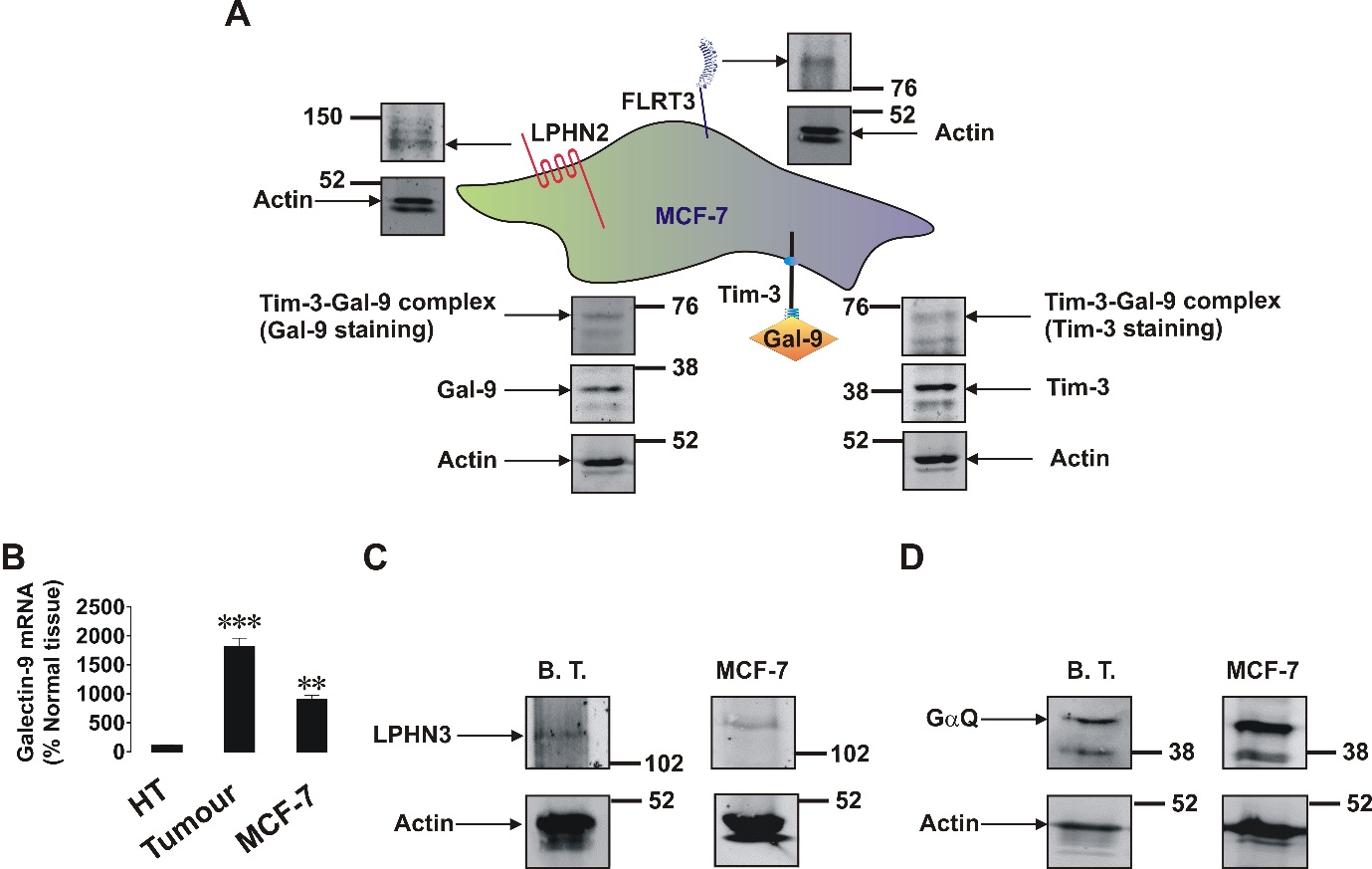
**

**Supplementary Figure 1. Expression of various FLRT3/LPHN/Tim-3/galectin-9 pathway components in MCF-7 breast cancer cells and primary human breast tumour cells. (A)** Expression of a variety of the pathway components was measured in MCF-7 cells and primary breast tumour tissue lysates by Western blot analysis. Beta-actin was used as a housekeeping protein. **(B)** Levels of galectin-9 mRNA were compared in normal and healthy breast tissues as well as in MCF-7 cells and normalised against those of β-actin. **(C)** Expression of LPHN3 was detected in primary human breast tumour tissue lysates and MCF-7 cells. **(D)** Expression of GαQ was detected in primary human breast tumour tissue lysates and MCF-7 cells. Images are from one experiment representative of at least three which gave similar results. Data are the mean values ± SEM of five independent experiments; **, p<0.01 and *** when p<0.001 *vs* control (HT).

**
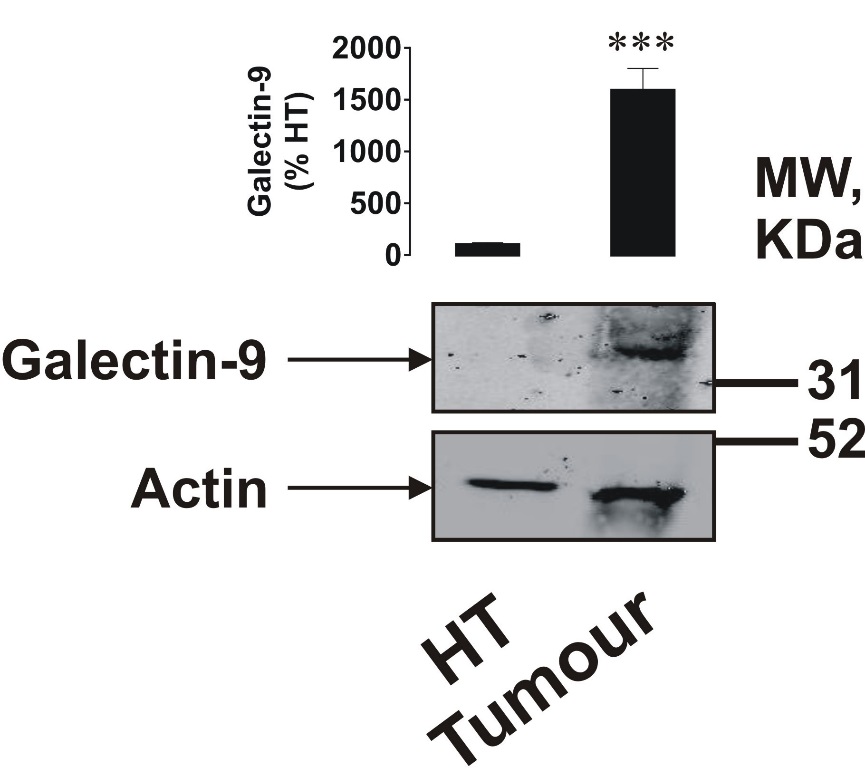
**

**Supplementary Figure 2. Expression of galectin-9 in primary human breast tumours.** Expression levels of galectin-9 were analysed in primary breast malignant tumours and healthy breast tissues (HT) of five patients (n=5) by Western blot using 10 % PAGE. Images are from one experiment representative of five which gave similar results. Other results are shown as mean values ± SEM. *** p<0.001 *vs* HT.

**
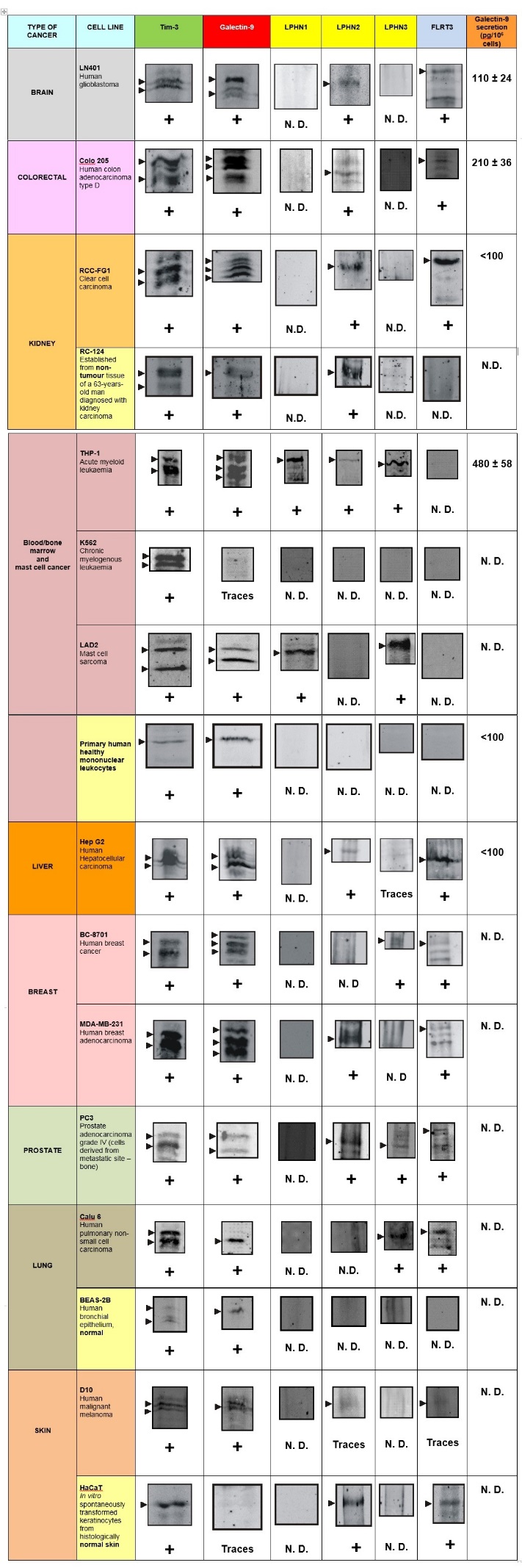
**

**Table 1 Expression of** **Tim-3, galectin-9, LPHNs 1, 2 and 3 as well as FLRT3 proteins in variety of cancer cell lines detectable by Western blot analysis.** **Tim-3:** lower band represents non-glycosylated protein, upper band(s), protein with differential levels of glycosylation; **Galectin-9**: multiple bands represent different isoforms of the same protein; FLRT3 – detectable between 80 and 95 kDa (upper band where applicable or the only visible band); another band (lower band; possibly extracellular domain) often appears at around 60 most likely reflecting levels of glycosylation in first two cases and proteolytic processing in the third. Traces – detectable expression which requires loading of >100 µg protein per well.


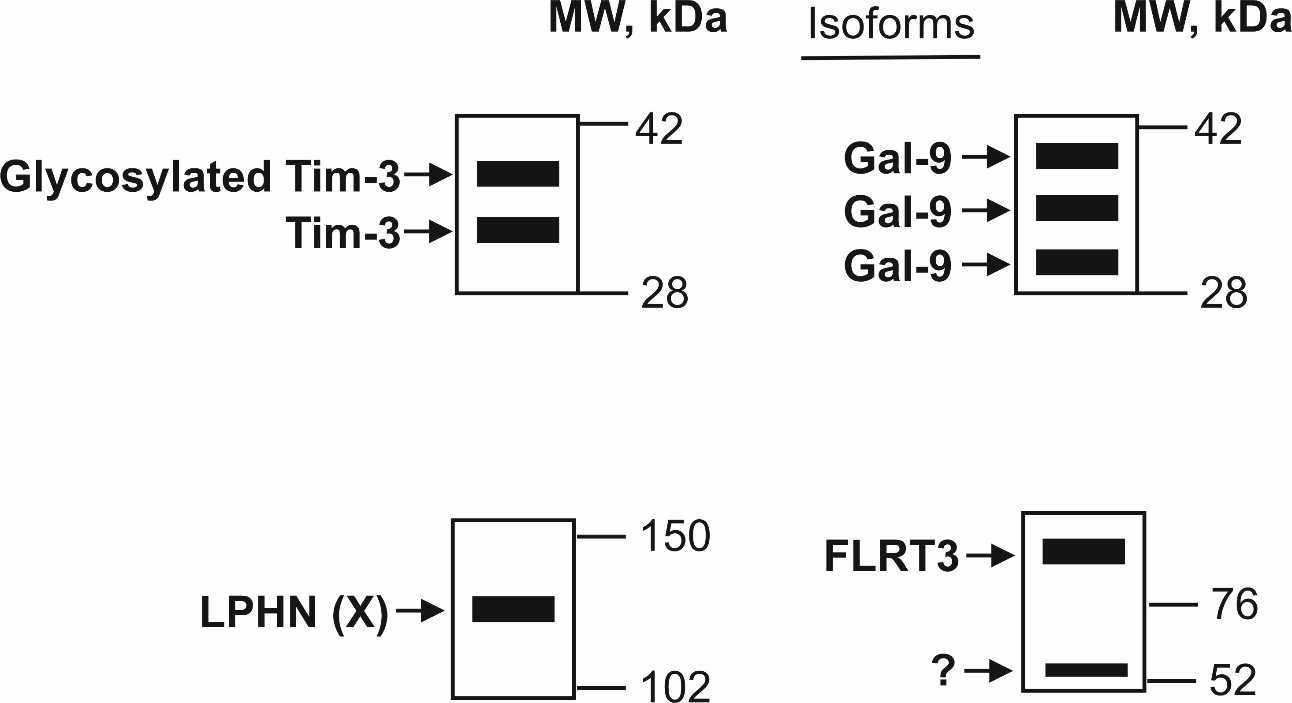


Scheme annotating Western blot images shown in the table.

|  |  |
| --- | --- |
|  |  |
|  | This colour is used to indicate non-malignant human cells |
